# Proteome Profile of Swine Testicular Cells Infected with Porcine Transmissible Gastroenteritis Coronavirus

**DOI:** 10.1371/journal.pone.0110647

**Published:** 2014-10-21

**Authors:** Ruili Ma, Yanming Zhang, Haiquan Liu, Pengbo Ning

**Affiliations:** 1 College of Veterinary Medicine, Northwest Agriculture & Forestry University, Yangling, Shaanxi, China; 2 College of Life Sciences, Northwest Agriculture & Forestry University, Yangling, Shaanxi, China; 3 School of Computer Science and Engineering, Xi’an Technological University, Xi’an, Shaanxi, China; University of Berne, Switzerland

## Abstract

The interactions occurring between a virus and a host cell during a viral infection are complex. The purpose of this paper was to analyze altered cellular protein levels in porcine transmissible gastroenteritis coronavirus (TGEV)-infected swine testicular (ST) cells in order to determine potential virus-host interactions. A proteomic approach using isobaric tags for relative and absolute quantitation (iTRAQ)-coupled two-dimensional liquid chromatography-tandem mass spectrometry identification was conducted on the TGEV-infected ST cells. The results showed that the 4-plex iTRAQ-based quantitative approach identified 4,112 proteins, 146 of which showed significant changes in expression 48 h after infection. At 64 h post infection, 219 of these proteins showed significant change, further indicating that a larger number of proteomic changes appear to occur during the later stages of infection. Gene ontology analysis of the altered proteins showed enrichment in multiple biological processes, including cell adhesion, response to stress, generation of precursor metabolites and energy, cell motility, protein complex assembly, growth, developmental maturation, immune system process, extracellular matrix organization, locomotion, cell-cell signaling, neurological system process, and cell junction organization. Changes in the expression levels of transforming growth factor beta 1 (TGF-β1), caspase-8, and heat shock protein 90 alpha (HSP90α) were also verified by western blot analysis. To our knowledge, this study is the first time the response profile of ST host cells following TGEV infection has been analyzed using iTRAQ technology, and our description of the late proteomic changes that are occurring after the time of vigorous viral production are novel. Therefore, this study provides a solid foundation for further investigation, and will likely help us to better understand the mechanisms of TGEV infection and pathogenesis.

## Introduction

Porcine transmissible gastroenteritis coronavirus (TGEV) is an animal coronavirus that causes severe gastroenteritis in young TGEV-seronegative pigs. Various breeds of pigs, regardless of age, are susceptible to TGEV; however, the mortality rate for piglets under 2 weeks of age is the highest, reaching almost 100%. Diseased pigs often present with vomiting, dehydration, and severe diarrhea. Further, the disease is known to affect pigs in many countries throughout the world and an outbreak can cause enormous losses in the pig industry [1], [2]. The pathogen, TGEV, which belongs to the *Alphacoronavirus* genus of the *Coronavirinae* subfamily within the family *Coronaviridae*, is an enveloped, non-segmented, single-stranded positive-sense RNA virus [3], [4]. The envelop, core, and nucleocapsid of the TGEV virion contain four major structural proteins: the nucleocapsid (N) protein, the membrane (M) glycoprotein, the small envelope (E) protein, and the spike (S) protein [5]. The tropism and pathogenicity of the virus are influenced by the S protein, which has four major antigenic sites, A, B, C, and D, with site A being the major inducer of antibody neutralization [3], [5]. The M protein, which plays a central role in virus assembly by interacting with viral ribonucleoprotein (RNP) and S glycoproteins [6], is embedded within the virus membrane and interacts with the nucleocapsid, forming the core of TGEV virion. In addition, the N-terminal domain of the M protein is essential for interferon alpha (IFN-α) induction [7], which is involved in the host’s innate immune response. The E protein, a transmembrane protein that acts as a minor structural component in TGEV and affects virus morphogenesis, is essential for virion assembly and release [8].

TGEV RNA, along with the N protein, is infectious and invades the organism through the digestive and respiratory tracts, resulting in infection of the small intestinal enterocytes, villous atrophy, and severe watery diarrhea. These changes in intestinal health are known to be important during the pathogenesis of TGEV infection [9]. Furthermore, corresponding to these pathologic changes observed in vivo, TGEV can also propagate and cause cytopathic effects (CPEs) in multiple types of cultured cells, such as swine testicular (ST) cells, PK-15 cells, and villous enterocytes. Notably, ST cells are more susceptible to TGEV, and higher levels of virus replication have been observed in this cell line [10], [11].

The full RNA genome of TGEV is approximately 28.5 kb in length and has a 5′-cap structure and a poly(A) tail at the 3′ end. The 9 open reading frame (ORF) genes included in the TGEV genome are arranged in the following order 5′-la- lb-S-3a-3b-E-M-N-7-3′. The first gene at the 5′ end consists of two large ORFs, ORF la and ORF lb, which constitute the replicase gene, known for its RNA-dependent RNA-polymerase and helicase activities, as well as other enzymes, such as endoribonuclease, 3′–5′exoribonuclease, 2′-O-ribose methyltransferase, ribose ADP 1” phosphatase, etc. [12]. ORF2, ORF4, ORF5, and ORF6 encode the S, E, M, and N proteins, respectively, while ORF3a, ORF3b, and ORF7 encode non-structural proteins [13]. Some investigators have suggested that ORF3 may be related to viral virulence and pathogenesis [12], while ORF7 may interact with host cell proteins and play a role in TGEV replication [14]. In fact, a recent study indicates that plasmid-transcribed small hairpin (sh) RNAs targeting the ORF7 gene of TGEV is capable of inhibiting virus replication and expression of the viral target gene in ST cells in vitro [15]. Although we have some knowledge concerning the translation and function of these viral proteins, the interactions that occur between these proteins and host cell proteins are not fully understood.

Importantly, recent advances in proteomic technology have allowed for more in depth investigation of virus-host interactions, and different techniques have been successfully applied to identify altered proteins in infected host cells and tissues. For example, Sun et al. [16] have identified 35 differentially expressed proteins in PK-15 cells infected with classical swine fever virus (CSFV) using two-dimensional polyacrylamide gel electrophoresis (2D PAGE) followed by matrix-assisted laser desorption-ionization time-of-flight tandem mass spectrometry (MALDI-TOF-MS/MS). In addition, two-dimensional fluorescence difference gel electrophoresis (2D-DIGE) and MS/MS proteomic approaches have been applied to characterize protein changes occurring in host cells in response to porcine circovirus type 2 (PCV2) infection [17]. The same methods have also been studied for many other pathogenic animal viruses, including porcine reproductive and respiratory syndrome virus (PRRSV) [18], coronavirus infectious bronchitis virus (IBV) [19], severe acute respiratory syndrome-associated coronavirus (SARS-CoV) [20], and TGEV [21]. However, these conventional approaches based on 2D gel electrophoresis are not suitable for detecting low abundance, hydrophobic, or very acidic/basic proteins. On the other hand, the isobaric tags for relative and absolute quantitation (iTRAQ) technique, in association with liquid chromatograph (LC), is a more advanced method for proteomic research, and is capable of detecting a much larger number of proteins, even those with low abundance, in addition to identifying and quantifying the proteins simultaneously [22]. To this end, Lu et al. [23] previously used the iTRAQ method to identify 160 significantly altered proteins in pulmonary alveolar macrophages (PAMs) infected with PRRSV. Similarly, this method has been used to investigate influenza virus infection in primary human macrophages [24], human immunodeficiency virus 1 (HIV-1) infection in CD4^+^ T cells [25], and Epstein–Barr virus (EBV) infection in nasopharyngeal carcinoma cell line [26].

Here, we report the first differential proteomic analysis of TGEV-infected and uninfected ST cells using iTRAQ labeling followed by 2D-LC-MS and bioinformatic analyses. The proteomic data obtained in this study will help to enhance our understanding of the host response to TGEV infection, but also provide new insights on the mechanisms of disease onset.

## Materials and Methods

### Cell culture and viral replication

ST cells were obtained from the American Type Culture Collection (ATCC). The cells were cultured in high-glucose Dulbecco’s modified Eagle’s medium (DMEM; GIBCO, UK) containing 1% L-glutamine and 10% fetal bovine serum (FBS) (Hyclone, Logan, UT) at 37°C in 5% CO_2_. Culture medium was replaced two to three times per week. The TGEV TH-98 strain was isolated from a suburb of Harbin, Heilongjiang province, China. The virus was propagated in ST cells and preserved at −70°C in our laboratory.

### TGEV infection

The monolayer of confluent ST cells was dispersed with 0.25% trypsin and 0.02% ethylenediaminetetraacetic acid (EDTA) and seeded in 6-cm cell culture flasks. After a 24 h incubation period, the culture medium was removed and the ST cells were washed with phosphate buffered saline (PBS, pH 7.4). The cells were then infected with the TGEV TH-98 strain at a 50% tissue culture infectious dose (TCID_50_) of 1×10^3.53^ viruses per well, with absorption for 2 h at 37°C. Maintenance medium (DMEM medium supplemented with 2% FBS) was then added to the cells. A mock group of ST cells that were not infected with TGEV was used as a negative control for each of the following experiments. Three replicates of virus-infected and mock-infected cultures with different passage numbers were prepared at each time point. The morphological changes were observed under the light microscope at 24, 40, 48, and 64 hours post infection (hpi).

### Reverse transcription polymerase chain reaction (RT-PCR) and real time quantitative PCR (qRT-PCR)

To determine the extent of TGEV infection, conventional RT-PCR and qRT-PCR assays were performed to detect the viral N gene. Monolayers of ST cells were infected with TGEV as described above. Cells were collected from 24 to 80 hpi at 8 h intervals, and the total RNA of the infected cells was extracted using Trizol (Invitrogen). RNA samples were reverse-transcribed using PrimeScript RT reagent Kit (Takara Bio, Dalian, China), according to the manufacturer’s instructions. The RT reaction was incubated at 37°C for 15 min followed by 85°C for 5 s. A mixture of oligo dT primers and random 6 mers was used in the RT step. The cDNA was stored at −20°C until further use.

PCR was performed for the TGEV N gene in a 25 µl reaction mixture containing 1 µl of the cDNA, 0.5 µl of each forward (F) and reverse (R) primer, 12.5 µl of Premix Taq (Takara Bio, Dalian, China), and 10.5 µl DEPC water, starting with a 5 min denaturation at 95 C followed by 32 cycles of 30 s denaturation at 95 C, 30 s annealing at 56 C, and 40 s extension at 72 C. A final extension step was carried out at 72 C for 10 min. RT-PCR products were resolved on a 15 g/L agarose gel. The following PCR primers were used in this study: TGEV N (F, 5′-GAGCAGTGCCAAGCATTACCC-3′ and R, 5′-GACTTCTAT CTGGTCGCCATCTTC-3′) and β-actin (F, 5′-GCAAGGACCTCTACGCCAA-3′ and R, 5′-CTGGAAGGTGGACAGCGAG-3′).

The mRNA expression level of the TGEV N gene was quantified using a SYBR Green assay on a Bio-Rad iQ5 real time PCR detection system as described previously [27]. We used the same primers listed above for qRT-PCR. Reactions were carried out in 50 µl volumes containing 0.5 µl of 20 × SYBR Green I, 2 µl of cDNA template, 1 µl of each F and R primer, 25 µl of 2 × PCR buffer, and 20.5 µl DEPC water. The cycling conditions were 94°C for 4 min, followed by 35 cycles of 94°C for 20 s, 60°C for 30 s, 72°C for 30 s, and then a final extension of 10 min at 72°C. The relative gene expression was determined with the 2^(−ΔΔCt)^ method [28], and the tests were performed in triplicate.

### Protein isolation, digestion, and labeling with iTRAQ reagents

Following ST cell infection, cells were collected at 48 and 64 hpi by centrifugation at 3,000 rpm for 5 min at 4°C, washed twice with PBS, and 1 mL of iTRAQ lysis solution (8 M urea, 1% (w/v) dithiothreitol (DTT)) containing protease inhibitor was added. Then, the cells were put in an ice bath and broken up by sonication. The solution was then mixed for 30 min at 4°C. The soluble protein fraction was harvested by centrifugation at 40,000 × *g* for 30 min at 4°C and the debris was discarded. The protein concentration was determined with the Bradford protein assay (2-D Quant Kit, Bestbio, China). A 100 µg aliquot of protein from each sample was reduced, alkylated, and trypsin-digested as described in the iTRAQ protocol (AB Sciex, American), followed by labeling with the 4-plex iTRAQ Reagents Multiplex Kit according to the manufacturer’s instructions (AB Sciex, American). Two virus-free samples at 48 h and 64 h were labeled with iTRAQ tags 114 and 115, while two TGEV-infected samples at 48 h and 64 h were labeled with tags 116 and 117. The labeled digests were then pooled, dried using a vacuum freeze drier (Christ RVC 2−25, Germany), and preserved at −20°C for later use.

### 2D LC-MS/MS analysis

The combined peptide mixtures were separated by reversed phase high-performance liquid chromatography (HPLC) (Ekspert ultraLC 100, AB Sciex, USA) on a Durashell-C18 reverse phase column (4.6 mm × 250 mm, 5 µm 100 Å, Agela). The mobile phases used were composed of 20 mM ammonium formate (pH 10) in water (labeled mobile phase A) and 20 mM ammonium formate (pH 10) in acetonitrile(ACN) (mobile phase B). The flow rate was 0.8 mL/min, and the elutant was collected into 48 centrifuge tubes at each minute after the first 5 min. Each aliquot was then dried by vacuum freezing.

The peptides were then analyzed with a nanoflow reversed-phase liquid chromatography-tandem mass spectrometry (nano-RPLC-MS/MS) system (TripleTOF 5600, AB Sciex, USA). The above 48 tubes were merged into 10 components dissolved in 2% ACN and 0.1% formic acid (FA), then centrifuged at 12,000 × *g* for 10 min. The supernatant (8 µl) was used for loading at a rate of 2 µl/min, with a separation rate of 0.3 µl/min. The mobile phase A used in this analysis was composed of 2% ACN and 0.2% FA, while mobile phase B was composed of 98% ACN and 0.1% FA. The following MS parameters were utilized: source gas parameters (ion spray voltage: 2.3 kV, GS1∶4, curtain gas: 30 or 35, DP: 100 or 80); TOF MS (m/z: 350–1250, accumulation time: 0.25 s); and product ion scan (IDA number: 30, m/z: 100–1500, accumulation time: 0.1 s, dynamic exclusion time: 25 s, rolling CE: enabled, adjust CE when using iTRAQ reagent: enabled, CES: 5).

### Data analysis and bioinformatics

Protein identification and quantification were performed with the ProteinPilot software (version 4.0, AB Sciex) using the Paragon algorithm. Each MS/MS spectrum was searched against a database of *Sus scrofa* protein sequences (NCBI nr, released in March 2011, downloaded from ftp://ftp.ncbi.nih.gov/genomes/Sus_scrofa/protein/). The following search parameters were used: iTRAQ 4-plex (peptide labeled), cysteine alkylation with methyl methanethiosulfonate(MMTS), trypsin digestion, biological modifications allowed, a thorough search, a detected protein threshold of 95% confidence (unused Protscore ≥1.3), and a critical false discovery rate (FDR) of 1%. The peptide and protein selection criteria for relative quantitation were performed as described previously, whereby only peptides unique for a given protein were considered [29]. In addition, proteins with an iTRAQ ratio higher than 20 or lower than 0.05 as well as proteins in reverse database were removed [30].

To assign enriched Gene Ontology (GO) terms to the identified proteins, the differentially expressed proteins identified from iTRAQ experiments and all of the 4,112 measured proteins were classified based on their GO annotations using QuickGO (http://www.ebi.ac.uk/QuickGO/), with UniProt ID (http://www.uniprot.org/?tab=mapping) as the data source. GO enrichment analysis of the differentially regulated proteins was evaluated using all of the 4,112 quantified proteins as background with hypergeometric distribution [31]. Categories belonging to biological processes, molecular functions, and cellular components that were identified at a confidence level of 95% were included in the analysis. The protein-protein interaction network for a select group of proteins was analyzed using the STRING 9.1 database (http://string-db.org/). Network analysis was set at medium confidence (STRING score >0.4).

### Western blot analysis

Following ST cell infection with TGEV, the culture medium was removed after incubating for 48 h and 64 h; then, the cells were washed with cold PBS and collected after centrifugation at 3,000 rpm for 10 min. Cells were then lysed in RIPA lysis buffer with protease inhibitors (Applygen Technologies Inc., China). Cellular debris was removed by centrifugation at 12,000 × *g* for 5 min at 4°C, and the protein concentration was measured by Coomassie blue G250 staining. An equal amount (20 µg) of cell lysate from each sample was separated using 10% SDS-PAGE and then transferred to polyvinyl difluoride (PVDF) membranes (Millipore, Bedford, USA). The PVDF membranes were then blocked with 5% (w/v) de-fatted milk powder dissolved in tris buffered saline and tween 20 (TBST) buffer (150 mM NaCl, 50 mM Tris, 0.05% Tween 20) for 1 h at 37°C. After blocking, membranes were incubated with anti-glyceraldehyde 3-phosphate dehydrogenase (GAPDH) mouse monoclonal antibody (1∶3000; Western Biotechnology, China), anti-heat shock protein 90 alpha (Hsp90α/HSP90AA1) antibody (1∶300; Abcam, Cambridge, UK), anti-caspase 8 antibody (1∶300; Abcam, Cambridge, UK), or anti-transforming growth factor β 1 (TGF-β1/TGFB1) antibody (1∶300; Abcam, Cambridge, UK) overnight at 4°C, followed by HRP-conjugated secondary antibody (1∶5000; Western Biotechnology, China) for 1.5 h at 37°C. The membranes were then washed four times in TBST buffer for 5 min each time. Protein band detection was performed using ECL reagents (Applygen Technologies Inc., China), and the band intensities were analyzed using Labworks 4.6 software.

## Results

### Confirmation of TGEV infection in ST Cells

**Figure 1:**
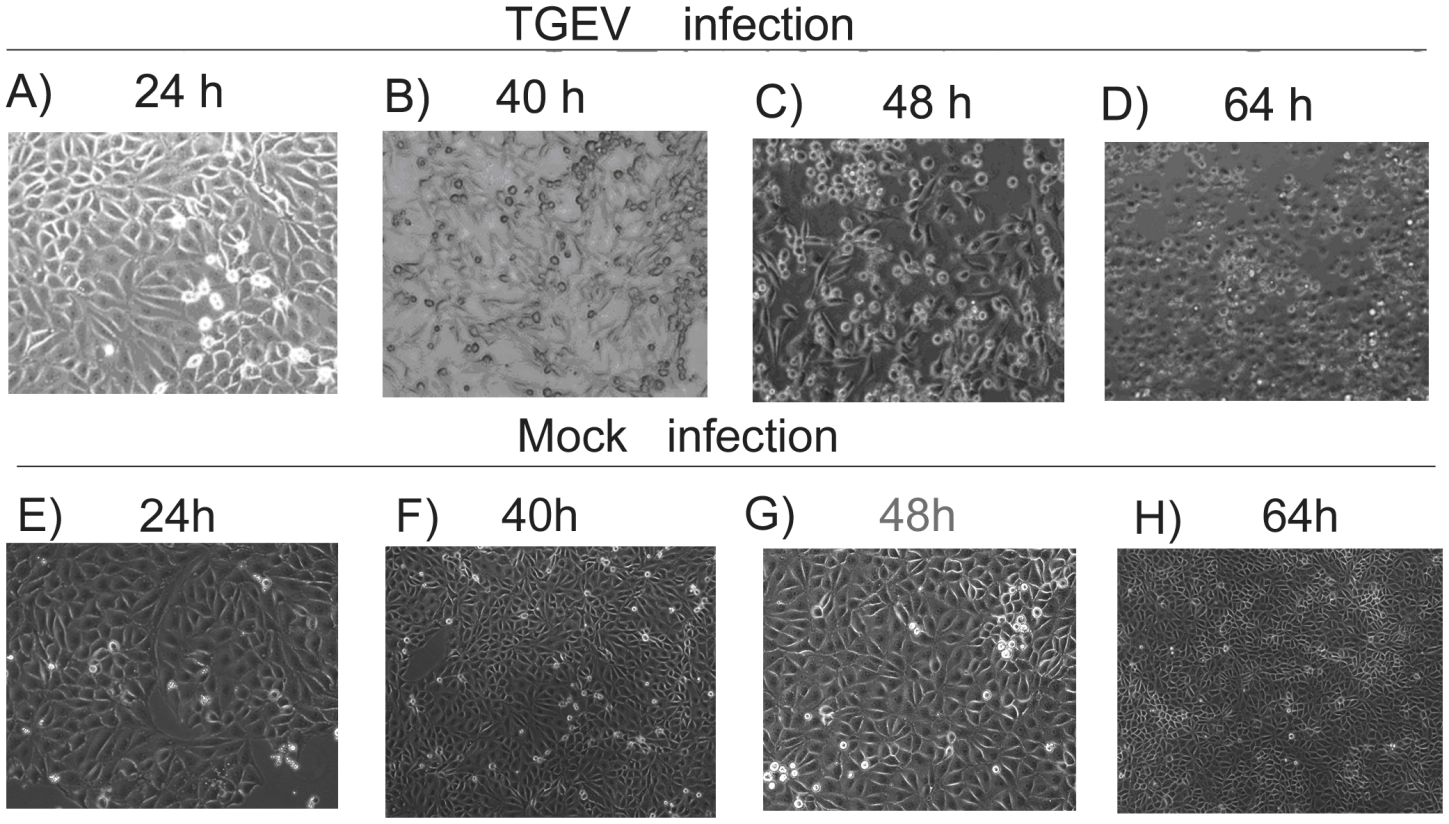
Morphological changes in TGEV-infected cells. ST cells were seeded into 6-cm culture plates, infected with TGEV, and the cytopathic effects (CPEs) were imaged at 24 (A), 40 (B), 48 (C), and 64 (D) hours following infection. Images of non-infected cells (mock infection) are shown for comparison at each time point (E, F, G, H).

**Figure 2:**
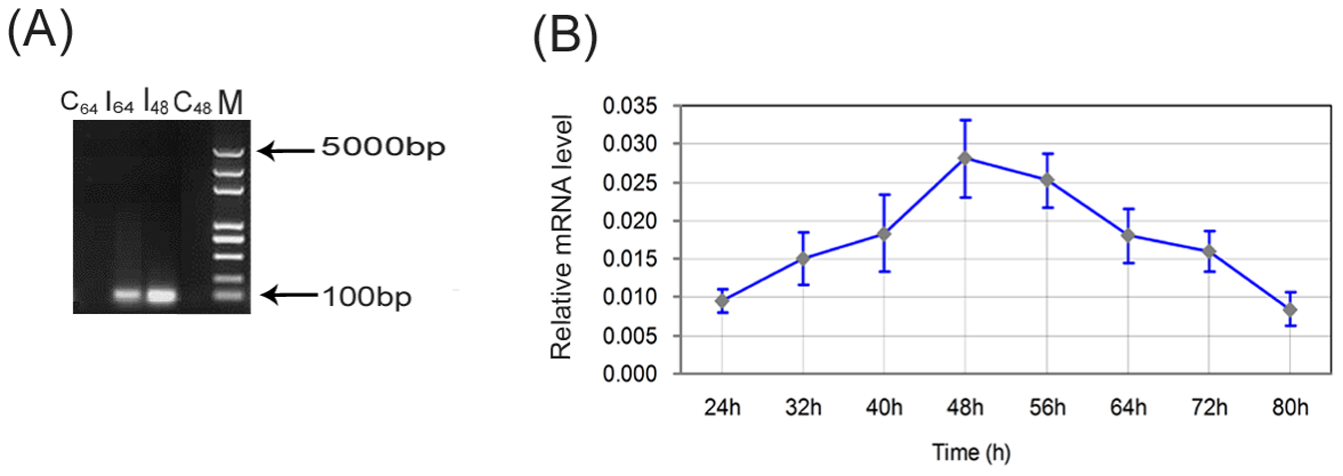
Validation of TGEV virus infection of ST cells. (A) RT-PCR validation of TGEV infection in ST cells at 48 hpi (I_48_) and 64 hpi (I_64_) compared to the control at 48 h (C_48_) and 64 h (C_64_). A marker (M) was used to identify fragment size. (B) qRT-PCR analysis of changes in TGEV mRNA expression levels in the ST cells over time. The changes in mRNA expression level at the various time points is indicated, and show that the expression level of TGEV increased gradually, reaching a peak at 48 h, then decreased dramatically. Values are the means of three repeated experiments. The error bars in the graphs represent the standard deviation.

After introducing TGEV into the ST cells, we observed the induction of typical CPEs, including cell rounding, swelling, granular degeneration of the cytoplasm, cell detachment, and severely diseased cell morphology, from 40 to 64 h after inoculation (Figure 1 A–D) compared to the non-infected control cells (Figure 1 E–H). Virus infection at 48 and 64 h was also confirmed by RT-PCR detection of the viral N gene in the sample (Figure 2A).

### Dynamic changes in viral gene expression in infected cells

To further identify the extent of TGEV infection, the mRNA expression levels of viral genes in infected cells were determined using qRT-PCR. Comparative threshold (Ct) cycle values in three independent experiments were calculated and the results indicated that the average Ct value for the TGEV N gene ranged from 25.2 to 27.5. Correspondingly, the average Ct value observed for the β-actin control gene ranged from 19.6 to 21.0. The relative expression of TGEV N mRNA was calculated using the 2^(–ΔΔCT)^ method [28], and the change in expression at each time point is indicated in Figure 2B. These data show that, following infection, the viral mRNA levels increased gradually over time, and reached a peak at 48 hpi. Following this time point, the viral mRNA levels appear to decrease.

### Protein identification by MS

**Figure 3:**
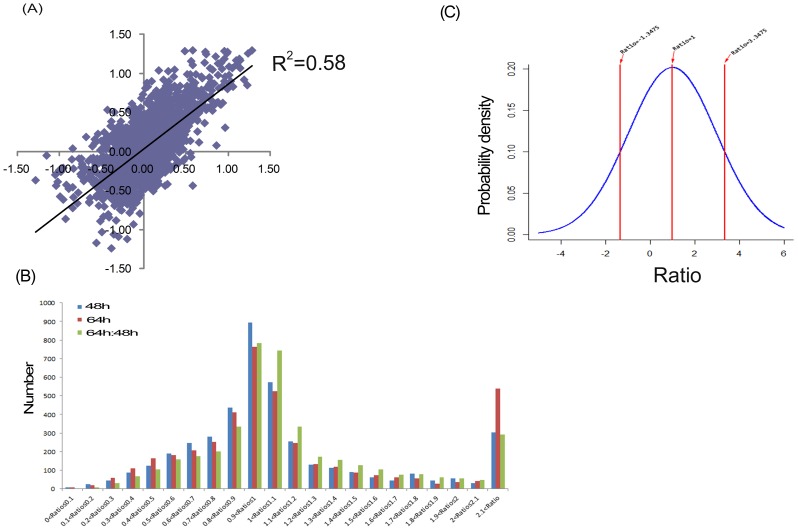
Results of the iTRAQ ratios analysis. (A) A scatter plot showing the correlation between the log_10_ infection/mock ratios at 48 hpi and 64 hpi for the 4,112 reliably quantified proteins in the iTRAQ experiment. Linear regression analysis shows that correlation (*R* ^2^) was 0.58, with a p-value less than 0.05. (B) Histograms showing the distribution of protein ratios identified at 48 and 64 hpi. (C) The distribution range of differentially expressed proteins identified at 48 hpi. iTRAQ ratios higher than 3.3475 (p = 0.975) or lower than −1.3475 (p = 0.025) were defined as statistically significant.

In the infected ST cells, a total of 29,214 peptides and 4,364 proteins were detected (Table S1); however, only 4,112 proteins were quantified reliably (Table S2). Notably, the abnormal proteins, such as the proteins with iTRAQ ratio higher than 20 or lower than 0.05, which are not quantifiable [30], were removed and only proteins with reasonable ratios across all channels were investigated further. Figure 3A depicts the scatter plots for the log_10_ 116/114 and log_10_ 117/115 ratios in the iTRAQ experiment. Linear regression analysis showed that correlation (*R* ^2^) was 0.58, with a p-value less than 0.05. These results suggest that the alterations in protein abundance due to virus infection were near-linear dependency between the two time points. In order to identify the proteins that were significantly different at each time point (infected/uninfected) or between the different time points, we analyzed the distribution of ratios for the identified proteins as shown in the Figure 3B. For the distribution range of the differentially expressed proteins identified at 48 hpi, shown in Figure 3C, a ratio higher than 3.35 or lower than −1.35 was defined as a statistically significant difference in protein expression. At 64 hpi, a ratio higher than 4.55 or lower than −2.15 was defined as a statistically significant difference in protein expression. According to analyses, the differentially expressed proteins identified were considered to show a significant upward or downward trend if their expression ratios were greater than 4.0 or less than 0.25 compared to the control group.

Using the criterion listed above, the expression of 146 proteins was significantly changed at 48 hpi (95 upregulated and 51 downregulated), while 219 proteins were significantly changed at 64 hpi (172 upregulated and 47 downregulated). Further, 72 proteins were identified to be significantly different between the two time points (54 upregulated and 18 downregulated), resulting in a total of 316 unique proteins being significantly altered during TGEV infection, including 162 predicted proteins (Table S3 and Table 1 (excluding the predicted proteins)). Because the current pig genome database is poorly annotated compared to the human genome database, there were numerous proteins that were unassigned or uncharacterized, resulting in a large number of predicted proteins in our analysis. However, our ability to detect the unannotated proteins by MS demonstrates that they do existence in this species, and additional research concerning their function is warranted.

**Table 1:** Differentially expressed proteins identified by iTRAQ analysis of ST cells infected with TGEV.

Accession number	Protein name	Gene symbol	Unused ProtScore	Infected/uninfected (48 h)		Infected/uninfected (64 h)	
				Ratio	P-value	Ratio	P-value
	**Upregulated proteins**						
gi|359811347	60 kDa heat shock protein, mitochondrial	–	139.52	3.16	0.00	5.86↑	0.00
gi|227430407	Keratin, type II cytoskeletal 8	KRT8	110.35	4.02↑	0.00	6.49↑	0.00
gi|347300243	Glutamate dehydrogenase 1, mitochondrial	GLUD1	102.28	1.79	0.18	4.17↑	0.00
gi|297591975	ATP synthase subunit alpha, mitochondrial	ATP5A1	96.61	1.17	0.67	4.66↑	0.00
gi|417515796	Hypoxia up-regulated protein 1 precursor	–	92.84	3.91	0.01	6.92↑	0.00
gi|349732227	Heterogeneous nuclear ribonucleoprotein M	–	89.74	7.66↑	0.00	9.64↑	0.00
gi|56748897	Heat shock 70 kDa protein 1B	HSPA1B	62.37	4.33↑	0.21	4.02↑	0.12
gi|47522630	Aspartate aminotransferase, mitochondrial precursor	GOT2	60.36	1.43	0.01	4.66↑	0.00
gi|387912908	Calreticulin	CALR	55.58	2.40	0.11	4.61↑	0.00
gi|346421378	Serpin H1 precursor	–	52.10	3.22	0.00	4.06↑	0.00
gi|2506849	Malate dehydrogenase, mitochondrial	MDH2	49.19	3.28	0.00	6.98↑	0.00
gi|148230268	Galectin-3	LGALS3	48.39	3.08	0.15	5.06↑	0.01
gi|417515899	2-oxoglutarate dehydrogenase, mitochondrial	–	45.01	2.99	0.02	5.25↑	0.00
gi|8745552	Voltage-dependent anion channel 1	VDAC1	43.46	6.19↑	0.01	11.59↑	0.00
gi|330417958	Phosphoenolpyruvate carboxykinase [GTP], mitochondrial	PCK2	42.89	1.96	0.09	5.75↑	0.00
gi|353468887	Signal transducer and activator of transcription 1	STAT1	42.79	1.80	0.33	6.98↑	0.00
gi|21264506	Succinyl-CoA ligase [GDP-forming] subunit beta, mitochondrial	SUCLG2	41.68	1.53	0.00	4.06↑	0.00
gi|47716872	Galectin-1	–	41.49	5.20↑	0.05	4.66↑	0.06
gi|342349346	Lon peptidase 1, mitochondrial	–	41.02	2.49	0.03	6.03↑	0.00
gi|210050415	Mx2 protein	Mx2	40.44	3.08	0.79	18.88↑*	0.00
gi|342349319	Calnexin precursor	–	37.71	4.79↑	0.00	6.14↑	0.00
gi|72535198	Histone H1.3-like protein	–	36.51	1.69	0.38	8.32↑*	0.12
gi|347300207	Nucleobindin-1 precursor	NUCB1	35.30	3.40	0.00	5.01↑	0.00
gi|347800693	Ferredoxin reductase	FDXR	33.41	1.56	0.05	4.57↑	0.00
gi|417515788	Prolow-density lipoprotein receptor-related protein 1 precursor	–	32.44	1.60	0.13	5.65↑	0.00
gi|297747350	FAT tumor suppressor homolog 1	–	32.04	6.08↑	0.00	7.24↑	0.00
gi|298104076	Enoyl-CoA hydratase, mitochondrial	–	30.50	2.21	0.23	6.55↑	0.00
gi|7939586	Dihydrolipoamide succinyltransferase	DLST	30.42	1.71	0.17	4.06↑	0.00
gi|7404364	Hydroxyacyl-coenzyme A dehydrogenase, mitochondrialPrecursor	HADH	29.29	1.92	0.00	5.50↑	0.00
gi|346644866	Coiled-coil-helix-coiled-coil-helix domain-containing protein 3, mitochondrial	CHCHD3	28.98	2.51	0.01	4.61↑	0.00
gi|47522814	Dihydrolipoyllysine-residue acetyltransferase component of pyruvate dehydrogenase complex, mitochondrial precursor	–	28.30	1.98	0.32	4.92↑	0.00
gi|6165556	Long-chain 3-ketoacyl-CoA thiolase	LCTHIO	26.88	3.98	0.00	7.59↑	0.00
gi|156720190	Mx1 protein	Mx1	26.26	3.80	0.98	19.41↑*	0.00
gi|47522770	Clusterin precursor	CLU	25.75	13.80↑	0.00	14.59↑	0.00
gi|347300323	Thioredoxin-dependent peroxide reductase, mitochondrial	PRDX3	24.24	2.72	0.00	6.92↑	0.00
gi|47522610	Succinyl-CoA:3-ketoacid coenzyme A transferase 1, mitochondrial precursor	OXCT1	23.94	1.72	0.22	6.37↑	0.00
gi|346986361	Electron-transfer-flavoprotein, alpha polypeptide	ETFA	22.41	2.42	0.34	5.55↑	0.00
gi|172072653	Lactadherin precursor	MFGE8	22.33	10.19↑	0.00	9.64↑	0.00
gi|56417363	Cathepsin D protein	–	21.95	0.54	0.0	2.38*	0.02
gi|87047636	ATP synthase H+-transporting mitochondrial F1 complex O subunit	ATP5O	21.83	1.11	0.66	7.66↑*	0.00
gi|89573851	Succinate dehydrogenase complex subunit B	SDHB	21.18	2.00	0.04	5.97↑	0.00
gi|5921142	Amyloid precursor protein	APP	20.29	13.43↑	0.00	15.14↑	0.00
gi|347658971	ATP synthase, H+ transporting, mitochondrial Fo complex, subunit d	–	20.26	3.34	0.01	9.82↑	0.00
gi|75052621	Transcription factor A, mitochondrial	TFAM	19.14	2.01	0.00	5.50↑	0.00
gi|312283580	Superoxide dismutase [Mn], mitochondrial	–	18.40	1.79	0.18	5.35↑	0.00
gi|6093657	Propionyl-CoA carboxylase beta chain, mitochondria	PCCB	17.92	2.33	0.13	6.85↑	0.00
gi|346716275	DnaJ homolog subfamily B member 11 precursor	DNAJB11	17.48	2.63	0.01	4.74↑	0.00
gi|118403762	Extracellular superoxide dismutase precursor	-	16.78	11.27↑	0.01	11.48↑	0.01
gi|150251019	Adenylate kinase 3-like 1	AK3L1	15.85	2.05	0.12	4.06↑	0.00
gi|158517860	Thymosin beta-10	TMSB10	13.45	6.25↑	0.30	7.52↑	0.30
gi|47522698	Cathepsin L1 precursor	CTSL	12.72	4.45↑	0.01	5.11↑	0.01
gi|329744622	Low-density lipoprotein receptor precursor	LDLR	12.69	3.50	0.04	4.06↑	0.01
gi|346644882	Reticulocalbin 2, EF-hand calcium binding domain precursor	RCN2	12.05	2.00	0.04	4.09↑	0.00
**gi|284519712**	**Caspase-8**	–	**11.38**	**7.11↑**	**0.13**	**16.14↑**	**0.00**
gi|211578396	Nitrogen fixation 1-like protein	LOC100156145	11.29	3.60	0.02	5.45↑	0.00
gi|346644830	Sulfide:quinone oxidoreductase, mitochondrial	SQRDL	10.92	1.87	0.38	4.53↑	0.02
gi|417515419	Semaphorin-3C precursor	–	10.76	4.92↑	0.08	3.94	0.24
gi|75064988	Syndecan-4	SDC4	10.29	18.88↑	0.00	19.59↑	0.00
gi|346716228	Histidine triad nucleotide-binding protein 2, mitochondrial isoform 2 precursor	HINT2	10.06	3.77	0.20	12.71↑	0.03
gi|85720739	Beta-enolase 3	ENO3	9.83	15.42↑	0.20	8.32↑	0.25
gi|223634702	Succinyl-CoA ligase [ADP/GDP-forming] subunit alpha, mitochondrial	SUCLG1	9.76	4.74 ↑	0.00	9.04↑	0.00
gi|4579751	130 kDa regulatory subunit of myosin phosphatase, partial	–	9.64	8.39↑	0.00	3.94	0.27
gi|76781337	ADAMTS1	ADAMTS1	9.61	6.79 ↑	0.00	7.38↑	0.00
gi|417515625	Interferon-induced protein with tetratricopeptide repeats 2	–	9.52	1.53	0.68	10.76↑*	0.00
gi|47522640	CD97 antigen	–	8.47	5.40↑	0.01	5.20↑	0.02
gi|55247591	Granulin precursor	GRN	8.43	12.71↑	0.00	14.59↑	0.01
gi|8347147	Inflammatory response protein 6	RSAD2	8.22	0.72	0.88	4.06↑*	0.00
gi|148234138	Cytochrome c oxidase subunit 6B1	COX6B	8.11	2.65	0.11	4.97↑	0.01
gi|343790890	Acyl-CoA dehydrogenase family, member 8	–	8.06	1.56	0.30	5.55↑	0.02
gi|9957597	Probable ATP-dependent RNA helicase DDX58	DDX58	7.83	1.22	0.52	8.02↑*	0.00
gi|347300255	DAZ-associated protein 1	DAZAP1	7.32	7.52↑	0.01	4.29↑	0.05
gi|148887343	ATP synthase subunit e, mitochondrial	ATP5I	7.00	1.11	0.97	4.21↑	0.01
gi|297632426	Signal sequence receptor, alpha	–	6.36	4.49 ↑	0.03	5.20↑	0.03
gi|6919844	Transforming growth factor-beta-induced protein ig-h3	TGFBI	6.12	4.92 ↑	0.01	3.56	0.20
gi|47523704	Double stranded RNA-dependent protein kinase	PKR	6.07	5.65↑	0.19	6.67↑	0.15
gi|339895859	Lipase, endothelial precursor	LIPG	5.14	4.06↑	0.04	3.25	0.05
gi|6226834	2'-5'-oligoadenylate synthase 1	OAS1	5.03	1.96	0.09	10.47↑*	0.01
gi|21636588	ATP synthase gamma subunit 1	–	4.61	2.78	0.16	4.49↑	0.05
gi|56392985	Asparagine-linked glycosylation 2	ALG2	4.31	2.65	0.30	4.57↑	0.23
gi|52346216	Fibroleukin precursor	FGL2	4.22	3.13	0.11	4.33↑	0.07
gi|154147577	Interferon-induced helicase C domain-containing protein 1	MDA5	4.20	2.09	0.78	6.67↑	0.06
gi|343098453	Chromatin target of PRMT1 protein	CHTOP	4.10	8.47↑	0.05	6.79↑	0.24
gi|343478189	Tubulin beta-2B chain	TUBB2B	4.04	5.25↑	0.30	5.40↑	0.24
gi|47523638	Nexin-1 precursor	PN-1	4.01	5.97↑	0.16	8.95↑	0.14
gi|346716354	Protein lunapark	–	4.00	10.76↑	0.17	7.94↑	0.23
gi|87047624	C-C motif chemokine 5	CCL5	3.80	5.35↑	0.31	18.71↑	0.12
gi|75056555	Integral membrane protein 2B	ITM2B	3.70	12.82↑	0.20	12.94↑	0.18
gi|264681460	Acyl carrier protein, mitochondrial	NDUFAB1	3.13	2.21	0.25	4.33↑	0.09
gi|456752927	Lectin, galactoside-binding, soluble, 3 binding protein	–	2.94	1.04	0.13	6.43↑*	0.06
gi|116175255	Regulator of differentiation 1	ROD1	2.79	2.68	0.23	4.29↑	0.14
gi|164664468	ATP synthase subunit epsilon, mitochondrial	ATP5E	2.74	3.66	0.14	14.06 ↑	0.02
gi|47522704	Vascular cell adhesion protein 1 precursor	–	2.72	3.56	0.11	6.79 ↑	0.02
gi|417515517	Solute carrier family 2,facilitated glucose transporter member 1	–	2.52	4.06↑	0.17	2.83	0.23
gi|346644790	Eukaryotic translation initiation factor 4E-binding protein 1	–	2.15	11.48↑	0.05	6.73↑	0.17
gi|346644828	Nuclear ubiquitous casein and cyclin-dependent kinases substrate	NUCKS1	2.01	5.70↑	0.24	3.60	0.37
gi|35208827	Macrophage colony-stimulating factor 1 precursor	MCSF alpha	2.01	6.37↑	0.24	7.73↑	0.21
gi|158726687	IGFBP-6	–	2.00	9.29 ↑	0.11	9.20↑	0.11
gi|146345485	Plasminogen	PLG	2.00	7.94 ↑	0.12	13.30↑	0.10
**gi|63809**	**Transforming growth factor beta-1**	**TGFB1**	**2.00**	**8.32↑**	**0.31**	**13.43↑**	**0.21**
gi|239504564	Claudin-4	CLDN4	1.97	4.92 ↑	0.27	8.63↑	0.16
gi|75049861	C-X-C motif chemokine 16	CXCL16	1.96	3.02	0.22	4.92↑	0.15
gi|158514029	ATP synthase lipid-binding protein, mitochondrial	ATP5G1	1.45	1.38	0.49	5.81↑*	0.34
gi|872313	Monocyte chemoattractant protein 1	CCL2	1.32	3.44	0.25	4.79↑	0.19
gi|81295909	Mitochondrial aldehyde dehydrogenase 2	ALDH2	34.88	0.72	0.14	3.16*	0.00
gi|224593280	Tyrosine-protein phosphatase non-receptor type 1	PTPN1	12.65	0.33	0.01	1,37*	0.11
gi|83415439	MHC class I antigen	PD1	7.05	0.45	0.43	3.13*	0.04
gi|148747492	Keratin, type II cytoskeletal 2 epidermal	KRT2A	6.68	0.67	0.98	3.40*	0.10
gi|75054309	N-acetylgalactosamine-6-sulfatase	GALNS	6.61	0.34	0.05	1.72*	0.11
gi|343791025	Lysosomal protective protein precursor	–	5.84	0.81	0.80	3.25*	0.05
gi|262204920	Peroxisomal trans-2-enoyl-CoA reductase	PECR	5.77	0.26	0.13	1.25*	0.32
gi|75063982	Alpha-crystallin B chain	CRYAB	4.92	0.37	0.19	3.40*	0.07
gi|456753359	Mevalonate (diphospho) decarboxylase, partial	–	4.01	0.26	0.44	1.41*	0.77
gi|343478257	Peptidase M20 domain containing 1	–	3.19	0.31	0.36	1.34*	0.69
gi|90024980	Peroxisomal enoyl coenzyme A hydratase 1	ECH1	17.09	0.79	0.88	3.37*	0.00
	**Downregulated proteins**						
gi|346986428	Heat shock 90kD protein 1, beta	HSPCB	130.10	0.70	0.52	0.21↓	0.00
gi|48675927	Tropomyosin alpha-3 chain	TPM3	91.83	0.53	0.01	0.20↓	0.00
gi|28948618	Chain A, structure of full-length annexin A1 in the presence of calcium	ANXA1	72.35	0.42	0.00	0.06↓*	0.00
**gi|6016267**	**Heat shock protein HSP 90-alpha**	**HSP90AA1**	**53.06**	**0.74**	**0.10**	0.19↓*	**0.00**
gi|47523720	Glucose-6-phosphate isomerase	GPI	50.00	0.54	0.00	0.18↓	0.00
gi|57527982	Radixin	RDX	44.08	0.53	0.00	0.22↓	0.00
gi|51702768	Peptidyl-prolyl cis-trans isomerase A	PPIA	41.51	0.75	0.35	0.24↓	0.00
gi|7650140	Gag-pol precursor	–	40.78	0.23↓	0.00	0.82	0.04
gi|262263205	Triosephosphate isomerase 1	TPI1	37.70	0.47	0.02	0.13↓	0.00
gi|1927	Cardiac alpha tropomyosin	TPM1	36.76	0.50	0.01	0.08↓*	0.00
gi|75074817	Peroxiredoxin-6	PRDX6	35.65	0.90	0.03	0.16↓*	0.00
gi|94962086	Aldo-keto reductase family 1 member C4	AKR1C4	34.49	0.12↓	0.00	0.37	0.00
gi|473575	Lactate dehydrogenase-B	LDHB	24.97	0.63	0.01	0.08↓*	0.00
gi|164414678	Alternative pig liver esterase	APLE	23.65	0.19↓	0.06	0.64	0.43
gi|343780946	D-dopachrome decarboxylase	DDT	19.05	0.15↓	0.26	0.60*	0.61
gi|347300176	Peroxiredoxin-2	PRDX2	24.03	0.74	0.30	0.24↓	0.01
gi|302372516	Heart fatty acid-binding protein	FABP3	23.79	0.60	0.00	0.23↓	0.00
gi|343887360	Proteasome (prosome, macropain) subunit, alpha type	–	21.65	0.42	0.00	0.25↓	0.00
gi|47522644	Acylamino-acid-releasing enzyme	APEH	20.10	0.38	0.01	0.15↓	0.00
gi|346716148	Importin-5	-	18.84	0.83	0.27	0.22↓	0.00
gi|47523046	Acyl-CoA-binding protein	DBI	18.36	0.44	0.05	0.23↓	0.00
gi|47523158	Glutathione S-transferase A2	–	15.78	0.31	0.00	0.09↓	0.00
gi|297591959	Farnesyl pyrophosphate synthase precursor	FDPS	15.29	0.67	0.32	0.16↓*	0.00
gi|56384247	Ribosomal protein L7	–	15.34	0.09↓	0.01	0.37	0.05
gi|347300398	Core histone macro-H2A.1 isoform 1	H2AFY	14.24	0.25↓	0.24	1.16*	0.58
gi|417515487	Collectin sub-family member 12	–	14.04	0.19↓	0.00	0.48	0.14
gi|94471896	signal transducer and activator of transcription 3	STAT3	13.42	0.21↓	0.00	0.58	0.14
gi|417515866	KIAA0196	–	12.91	0.39	0.00	0.14↓	0.00
gi|584724	Aminoacylase-1	ACY1	12.26	0.30	0.00	0.13↓	0.00
gi|158514030	60S ribosomal protein L14	RPL14	10.79	0.14↓	0.00	0.79*	0.85
gi|187606917	40S ribosomal protein S26	RPS26	6.00	0.19↓	0.07	0.45	0.15
gi|89257972	Protein phosphatase 1 catalytic subunit beta isoform	PPP1CB	5.27	0.11↓	0.25	0.61*	0.52
gi|417515889	FK506-binding protein 15	–	4.06	0.05↓	0.12	0.43*	0.25
gi|48474224	Scavenger receptor class B member 1	SCARB1	2.98	0.24↓	0.08	0.41	0.13
gi|83778524	Beta-tropomyosin	TPM2	2.55	0.28	0.21	0.07↓*	0.04
gi|298104074	Protein FAM54A	–	2.08	0.46	0.30	0.21↓	0.16
gi|342349308	Calmegin precursor	–	2.00	0.16↓	0.14	0.21↓	0.16
gi|1016311	Cytochrome P450 2C33v3, partial	–	1.96	0.10 ↓	0.11	0.41*	0.26
gi|346716298	Heterogeneous nuclear ribonucleoprotein G	RBMX	32.46	3.63	0.00	0.86*	0.41
gi|262263201	Squalene epoxidase	SQLE	2.02	1.37	0.67	0.34*	0.32

Note: *means the proteins significantly differed in expression level between 48 and 64 hpi. The three proteins verified by Western blot analysis are highlighted in bold. The corresponding ratios at each time point (infected/uninfected) are given. Gene symbols were retrieved from UniProt.

### GO enrichment analysis

Biological process-based enrichment analysis of the differentially expressed proteins revealed that six common GO terms were significantly enriched in this set of proteins (p<0.05). Thus, it appears that in TGEV-infected ST cells at 48 and 64 hpi there are expression changes in proteins that are related to cell adhesion, neurological system processes, extracellular matrix organization, locomotion, cell junction organization, and cell-cell signaling. Moreover, at the later time point, 64 hpi, our GO term analysis also indicated that a significant number of the differentially expressed proteins were related to cellular stress (p = 8.18E-4), generation of precursor metabolites and energy (p = 2.74E-3), cell motility (p = 6.71E-3), protein complex assembly (p = 4.69E-2), growth (p = 3.87E-2), developmental maturation (p = 1.53E-2), and immune system processes (p = 4.67E-2) (Table 2).

**Table 2:** Biological process-based GO term enrichment analysis.

GO term	Gene symbol or protein name (48 hpi)	P-value(48 hpi)	Gene symbol or protein name (64 hpi)	P-value(64 hpi)
**Cell adhesion**	MFGE8, CYR61, ITGA5, FN1, TGFBI, TGFB1, PN-1, CCL5, APP, PPP1CB, SCARB1	2.57E-3	MFGE8, CYR61, ITGA5, FN1, TGFB1, CALR, APP, TACSTD2, PN-1, Vascular cell adhesion molecule, CCL5	2.57E-3
Response to stress	NUDT9, CYR61, ITGA5, FN1, PLG, TGFB1, Extracellular superoxide dismutase precursor, CLU, CCL5, HSPA1B, PN-1, VDAC1	7.98E-1	CCL2, NUDT9, CYR61, ITGA5, FN1, LOC100516779, Mitochondrial heat shock 60 kDa protein 1, CCDC47, PLG, TGFB1, CALR, Extracellular superoxide dismutase precursor, OAS1, HSPA1B, PN-1, DDX58, VDAC1, RSAD2, HSP90AA1, HSPCB, DBI, PRDX2, PRDX3, CLU, CCL5, CXCL16, PRDX6	8.18E-4
Generation of precursor metabolites and energy	ENO3, SUCLG1, PPP1CB	6.51E-1	ENO3, TPI1, IDH3A, SUCLG1, LDHB, MDH2, GPI, SUCLG2, SDHB, DLST	2.74E-3
**Extracellular matrix organization**	CYR61, TGFB1, APP	1.22E-2	LGALS3, CYR61, TGFB1, APP	1.51E-3
**Locomotion**	TGFB1, APP, CCL5	1.22E-2	TGFB1, APP, CCL5, CXCL16	1.51E-3
Cell motility	STAT3, CYR61, ITGA5, TUBB2B, TGFB1, CCL5	2.07E-1	CCL2, TACSTD2, CYR61, ITGA5, TUBB2B, TGFB1, CALR, CCL5, CXCL16, DDX58	6.71E-3
**Cell-cell signaling**	ITPR3, APP, PN-1, VDAC1, CCL5	2.58E-2	GLUD1, APP, PN-1, VDAC1, CCL5	2.58E-2
**Neurological system process**	ITPR3, ITGA5, APP, VDAC1, PN-1	7.91E-3	ITGA5, APP, PN-1, VDAC1	3.34E-2
Protein complex assembly	H2AFY, SLAIN2, HIST1H2BF, TUBB2B, HIST1H2BJ, TMSB10, TGFB1, CLU, CCL5	2.66E-1	SLAIN2,HIST1H2BF,TUBB2B,HIST1H2BJ, TGFB1, TMSB10, RDX, CALR,CLU, CCL5, Histone H1.3-like protein, TFAM	4.69E-2
**Cell junction organization**	ITGA5, FN1, TGFB1	1.22E-2	ITGA5, FN1, TGFB1	1.22E-2
Growth	STAT3, CYR61, TGFB1, APP, PN-1	1.01E-1	COL9A1, CYR61, TGFB1, PN-1, APP, CXCL16	3.87E-2
Developmental maturation	APP	1.13E-1	ARCN1, APP	1.53E-2
Immune system process	TGFB1, CCL5	9.81E-1	CCL2, HSPCB, PRDX3, LOC100516779, TGFB1, CALR, OAS1, CCL5, CXCL16, DDX58, RSAD2	4.67E-2

Note: P-values were calculated in the hypergeometric test. Gene symbols were retrieved from UniProt. The significantly common processes affected are highlighted in bold.

To further investigate the localization pattern of these differentially expressed genes, a cellular component-based enrichment analysis was performed. At 48 hpi, we observed the significant enrichments in extracellular region (p = 1.29E-4), proteinaceous extracellular matrix (p = 1.62E-4), and extracellular space (p = 1.52E-2) (Table S4). In addition, 37 differentially expressed proteins were also significantly enriched (p = 8.65E-3) in mitochondrion at 64 hpi (Table S5).

The final step of our GO enrichment analysis consisted of investigating the mechanistic role these genes play in the cell. To do so, we performed a molecular function-based enrichment analysis. This analysis showed that two GO terms, unfolded protein binding (p = 2.67E-2) and transmembrane transporter activity (p = 3.55E-2), were significantly enriched at 64 hpi (Table S5). Further GO analysis of the differentially expressed proteins between the two time points indicated that there were no significant enriched terms.

### Protein–protein interaction analysis

**Figure 4:**
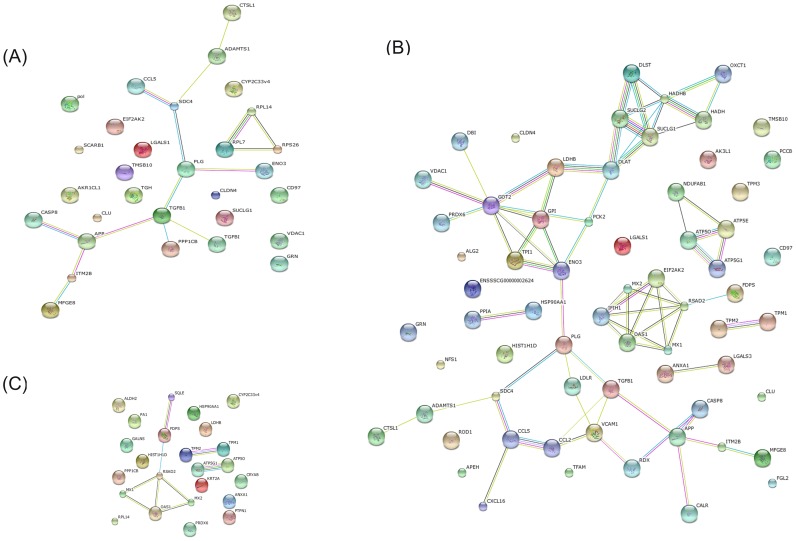
Protein-protein interaction network created using the STRING database. (A) Network of the differentially expressed proteins at 48 hpi. The network includes 30 nodes (proteins) and 15 edges (interactions). (B) Network of differentially expressed proteins at 64 hpi. The network includes 66 nodes and 70 edges. (C) Network of differentially expressed proteins between the two time points. The network includes 24 nodes and 9 edges. Network analysis was set at medium confidence (STRING score = 0.4). Seven different colored lines were used to represent the types of evidence for the association: green, neighborhood evidence; red, gene fusion; blue, co-occurrence; black, co-expression; purple, experimental; light blue, database; yellow, text mining.

In order to understand the interactions between TGEV and host cell proteins, we further analyzed the differentially expressed proteins by searching the STRING 9.1 database (http://string-db.org/) for protein-protein interactions (Figure 4). In this STRING analysis, the interactions (edges) of the submitted proteins (nodes) were scored according to known and predicted protein-protein interactions. We created three protein network maps: one for proteins changed significantly at 48 hpi (30 nodes and 15 edges; Figure 4A), one for proteins changed significantly at 64 hpi (66 nodes and 70 edges; Figure 4B), and one for the proteins that were significantly changed when the viral infection was prolonged from 48 to 64 h (24 nodes and 9 edges; Figure 4C). Notably, the protein network constructed for the 64 hpi time point is clearly much more extensive than the two other networks, and these protein-protein interactions suggest the existence of reported functional linkages. GO enrichment analysis for the STRING protein network at 64 hpi showed that several biological processes were significantly affected (p<0.05 based on the FDR correction) in this network, including the regulation of viral genome replication, the innate immune response, negative regulation of viral genome replication, positive and negative regulation of viral processes, and ATP biosynthetic processes (Table 3). However, at 48 hpi, the most enriched biological process was related to cell recognition during phagocytosis(p = 8.02E-1). In Figure 4C, we have shown that the majority proteins in these protein networks, such as radical S-adenosyl methionine domain containing protein 2 (RSAD2), Mx dynamin-like GTPase 1 (Mx1), 2′-5′-oligoadenylate synthetase 1 (OAS1), Mx dynamin-like GTPase 2 (Mx2), are involved in the innate immune response. These data suggest that some entirely different host proteins, interactions, or processes, including the immune response, were perturbed at these times during TGEV infection.

**Table 3:** List of the GO biological processes enriched for the proteins present in the STRING protein network.

GO biological process	P-value
Regulation of viral genome replication	1.33E-2
Innate immune response	1.35E-2
Negative regulation of viral genome replication	2.36E-2
Regulation of viral process	2.70E-2
Negative regulation of viral process	2.83E-2
ATP biosynthetic process	2.89E-2

Note: The significance of the GO biological process is derived from the network in Figure 4B and was determined using the FDR correction (p<0.05).

### Western blot confirmation of altered expression for three of the differentially expressed proteins

**Figure 5:**
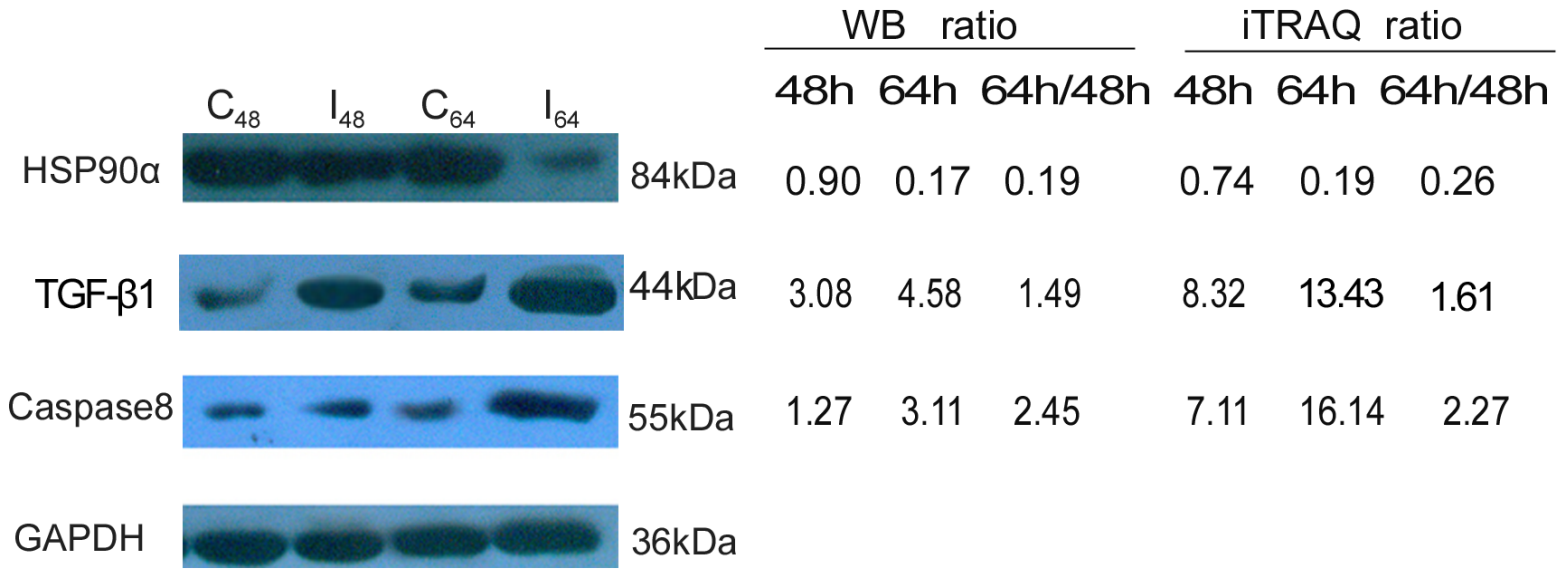
Western blot confirmation for three differentially expressed proteins (caspase-8, HSP90α, and TGF-β1). Following TGEV and mock infection of the ST cells, equal amounts of protein were separated by SDS-PAGE and transferred to PVDF membranes. The membranes were then probed with the specified antibody, and the identified bands were visualized. GAPDH was used as an internal control to normalize the quantitative data. The representative images shown are typical of two independent experiments. At 48 hpi (I_48_), integrated optical density (IOD) analysis showed an upregulation of caspase-8 (1.27 fold) and TGF-β1 (3.08 fold), but HSP90α was almost unchanged (0.90 fold). At 64 hpi (I_64_), we observed an upregulation in both caspase-8 (3.11 fold) and TGF-β1 (4.58 fold), but a 5.82 fold downregulation of HSP90α. The IOD was normalized against GAPDH.

To further confirm the proteomic data for three of the proteins, western blot analysis was performed to investigate the changes in the expression of HSP90α, caspase 8, and TGF-β1. The proteins were selected based on three criteria: 1) the expression of the protein was increased or decreased during TGEV infection according to our proteomics data; 2) the protein is known to be relevant during viral infection; and 3) each protein analyzed needs to be involved in a special biological process as determined by our GO enrichment analysis [32]. HSP90α, caspase 8, and TGF-β1 all filled these criteria and their protein expression was analyzed via western blot analysis of the cell lysate. As shown in Figure 5, the expression of HSP90α was significantly downregulated in TGEV-infected cells at 64 hpi, while the expression of caspase-8 was upregulated from 48 to 64 hpi in these cells. The expression of TGF-β1 was also significantly induced in TGEV-infected cells following infection. Thus, these results confirm the altered expression observed in the proteomic data for these three representative proteins during TGEV infection.

## Discussion

The interactions between a virus and a host cell during a viral infection are complex, involving numerous genes and signaling pathways. ST cells are known to be sensitive to TGEV, resulting in increased viral multiplication and CPEs [15]. In order to better understand the interactions between the host proteome and TGEV, we adopted an iTRAQ quantitative proteomic approach to investigate the altered cellular proteins of the ST cells during TGEV infection in vitro. Compared with the 2-DE and 2D-DIGE methods often used, the 2D-LC-MS/MS method utilized here provides more quantitative and qualitative information about the proteins, and can also detect membrane proteins, hydrophobic proteins, higher molecular weight proteins, and low-abundance proteins, which are often missed by other methods. iTRAQ also has more advantages compared to isotope-coded affinity tags (ICAT) and stable isotope labeling by amino acids in cell culture (SILAC) methods, which both allow multiple labeling and quantitation of four to eight samples simultaneously with high sensitivity [22], [33], [34]. Further, the iTRAQ technique has been widely used for quantitative proteomics, including protein expression analysis and biomarker identification [23]–[26], [35].

Prior to proteomic analysis, we determined which time points to investigate following infection by observing the morphological changes and analyzing viral gene expression dynamics in the TGEV infected cells. The results indicated that TGEV induced significant CPEs from 40 to 64 hpi in infected cells compared to the mock infected cells. At 40 hpi, less than 50% of the infected cells were morphologically altered, while at 48 hpi more than 80% infected cells showed rounding and granular degeneration. Further, the mRNA level of the viral N gene in ST cells continuously increased in the infected cells until 48 h, at which time we observed the highest viral replication level. At 64 hpi, the morphological effects observed were much more pronounced, characterized by even more cellular rounding and detachment. However, the mRNA levels of the viral N gene decreased rapidly from 48 to 64 h, a phenomenon we believe may be attributed to the host's immune response or a decrease in infected cell viability as the TGEV infection progressed. Based on our qRT-PCR and CPE analyses, we choose to more deeply investigate the proteomic changes occurring in the TGEV-infected ST cells at 48 hpi and 64 hpi using a 4-plex iTRAQ analysis.

In our analysis, we observed a statistically significant change in the expression of 316 proteins during TGEV infection in vitro. This number includes protein changes that were unique for a specific time point as well as those shared at these different time conditions. For example, the expression level of HSP90α expression was unchanged at 48 hpi, but decreased at 64 hpi, making this change unique for the latter time point. On the other hand, TGF-β1 was observed to increase at both of the time points, and was thus labeled a shared protein change. Moreover, the 316 altered proteins also includes proteins that changed from 48 hpi to 64 hpi, rather than one of these time points compared to non-infected cells. For example, mitochondrial aldehyde dehydrogenase 2 (ALDH2) and MHC class I antigen (PD1) were not changed at 48 or 64 hpi compared to the control group, but increased at 64 hpi compared with 48 hpi. We also observed a larger proteomic shift at 64 hpi compared to the 48 hpi time point in the infected ST cells.

Further, some proteins previously reported to play a role in virus-induced host cell death, such as caspase-8, caspase-3, caspase-9, and porcine aminopeptidase-N (pAPN) [36]–[38], were also identified using this iTRAQ technique. These caspase proteins are known to be involved in TGEV-induced cell apoptosis processes, while pAPN is the cell receptor for TGEV. Our results indicate that TGEV infection caused significant upregulation of caspase-8 expression at two time points (approximately 7-fold at 48 hpi and 16-fold at 64 hpi) in the virus-infected ST cells, and this change was verified by western blotting analysis. However, the expression of caspase-3, caspase-9, and pAPN was not significantly altered, indicating that the pathways involving these genes are not altered or that other proteins are compensating for their lack of change. In this regard, we identified an additional 15 proteins involved in cell death pathways that had significantly altered expression levels (p = 4.46E-2) (Table S6), including melanoma differentiation associated protein-5 (MDA5), monocyte chemoattractant protein 1 (CCL2), thioredoxin- dependent peroxide reductase, mitochondrial (PRDX3), peroxiredoxin-2 (PRDX2), predicted protein CYR61 (CYR61), keratin, type II cytoskeletal 8 (KRT8), predicted bcl-2-like protein 13 (BCL2L13), predicted integrin alpha-5 isoform 1 (ITGA5), TGF-β1, amyloid beta A4 protein (APP), clusterin (CLU), C–C motif chemokine 5 (CCL5), heat shock 70 kDa protein 1B (HSPA1B), alpha-crystallin B chain (CRYAB), voltage-dependent anion-selective channel protein 1 (VDAC1), all of which, with the exception of PRDX2 and BCL2L13 were upregulated at one or two time points. Regulation of cell death is known to be important for replication and pathogenesis in various coronaviruses [39], and we believe that further research on these proteins will lead to a better understanding of cell death regulation during TGEV infection.

In order to determine what other processes, in addition to cell death, were affected by TGEV infection, we performed a GO enrichment analysis for the different temporal conditions. This analysis indicated that six biological processes were significantly affected at 48 and 64 hpi, and the differentially expressed proteins involved in these processes were almost the same. The large overlap between the two time points suggests that some of the same sets of host proteins or processes were disturbed at these times. However, it is also likely that some processes were affected solely at one time point or the other. At 48 hpi, serine/threonine-protein phosphatase PP1-beta-catalytic subunit (PPP1CB), scavenger receptor class B member 1 (SCARB1), transforming growth factor-beta-induced protein ig-h3 (TGFBI), and predicted inositol 1,4,5-trisphosphate receptor type 3 (ITPR3) were uniquely altered, likely indicating changes in cell adhesion and/or cell-cell signaling processes. At 64 hpi, on the other hand, calreticulin (CALR), predicted tumor- associated calcium signal transducer 2-like (TACSTD2), vascular cell adhesion molecule, galectin-3 (LGALS3), glutamate dehydrogenase 1 (GLUD1), and C–X-C motif chemokine 16 (CXCL16) were uniquely changed, also indicating changes in cell adhesion and/or cell-cell signaling as well as extracellular matrix organization and locomotion. We believe that these uniquely altered proteins reflect changes in specific/specialized processes at each time point that are tightly linked to the temporal changes observed in the host cell morphology and gene/protein expression after TGEV infection.

The most significantly enriched GO category related to the differentially expressed proteins was stress, which included 12 differentially expressed proteins at 48 hpi and 27 different proteins at 64 hpi. The increased number of proteins association with this GO term at 48 hpi likely highlights the initial upregulation of the cellular stress response, while the higher number at 64 hpi indicates that the stress response to TGEV infection is likely more fully induced at this later stage. HSPs, also known as stress proteins, are often involved in the cellular response to stress, influencing changes in the state or activity of the cell or organism. HSP90, which has two isoforms (HSP90α and HSP90β), is one of the most abundant molecular chaperones that is induced in response to cellular stress, and it functions to stabilize proteins involved in cell growth and anti-apoptotic signaling [40]. The expression of HSP90α has been reported to play an important role in the replication of some viruses, such as Ebola virus (EBOV) [41], hepatitis C virus (HCV) [42], influenza virus [43], and Japanese encephalitis virus [44]. On the other hand, the reduction of HSP90β has been reported to decrease the correct assembly of human enterovirus 71 viral particles [40]. In this study, HSP90α and heat shock 90kD protein 1, beta (HSPCB/HSP90β) were significantly downregulated at 64 hpi in the TGEV-infected ST cells, but were unchanged at 48 hpi, indicating that they may play a similar role in TGEV infection. Interestingly, a member of the HSP70 protein family, heat shock 70 kDa protein 1B (HSPA1B), as well as mitochondrial 60 kDa heat shock protein (HSP60) were both upregulated in infected ST cells at 48 and/or 64 hpi. HSP60 is a mitochondrial chaperonin protein involved in protein folding and a number of extracellular immunomodulatory activities. Elevated expression of HSP60 is associated with a number of inflammatory disorders [45]. HSP70 plays an important role in multiple processes within cells, including protein translation, folding, intracellular trafficking, and degradation. A previous study has revealed that HSP70 is involved in all steps of the viral life cycle, including replication, and is highly specific in regards to viral response, differing from one cell to another for any given virus type [46]. For example, silencing HSP70 expression has been associated with an increase in viral protein levels, while an increase in HSP70 has been suspected to be the initial cellular response to protect against viral infection in rotavirus-infected cells [47]. Further, a recent study showed that HSP70 is an essential host factor for the replication of PRRSV as the silence of HSP70 significantly reduced PRRSV replication [48]. Our results provide new experimental evidence relating the expression of HSP90, HSP70, and HSP60 to TGEV infection, and we speculate that these proteins play a potential role in TGEV replication. Additional work is required to investigate the detailed role of these proteins during TGEV infection.

Furthermore, another significantly enriched GO process we observed that 11 significantly altered proteins was immune system processes. Most of these proteins were significantly upregulated at 64 hpi in response to the viral infection, while some were first upregulated at 48 hpi, including CCL5 and TGF-β1. Chemokines, such as CCL2, CCL5, and CXCL16, whose main function is macrophage recruitment and activation, are potentially involved in host-mediated immunopathology. A recent study showed that coronavirus infection of transgenic mice expressing CCL2 led to a dysregulated immune response without effective virus clearance and enhanced death [49]. In additional, TGEV-infection can induce the expression of proinflammatory genes, including CCL2, CCL5, and probable ATP-dependent RNA helicase DDX58 (DDX58/RIG-1), in cell culture and in vivo in the absence of viral protein 7 [50]. In this study, we observed an upregulation of CCL2, CCL5, CXCL16, TGF-β1, and DDX58 expression. TGF-β1 is a multifunctional cytokine, secreted from various cells, and, in immunology, it regulates cellular proliferation, differentiation, and other cellular functions for a variety of cell types, especially regulatory T cells [51]. Some research has indicated that **S**ARS-CoV papain-like protease (PLpro) increases TGF-β1 mRNA expression and protein production in human promonocytes [52]. Further, Gomez-Laguna et al. [53] inferred that the upregulation of the TGF-β may impair the host immune response during PRRSV infection by limiting the overproduction of proinflammatory cytokines necessary to decrease PRRSV replication. In response to viral infection, DDX58 plays important roles in the recognition of RNA viruses in various cells, and has been identified as a candidate for a cytoplasmic viral dsRNA receptor [54]. Further, upregulation of this gene activates cells to produce type I interferons, which may increase the antiviral status of cells to protect against viral infection. In this regard, we found that interferon-inducible antiviral proteins, RSAD2, OAS1, were also upregulated in the period of late infection, suggesting that many of the proteins identified in this study are associated with inflammation, IFN activation, and the innate immune response. Increased expression of these proteins may help the virus enter the cell as well as potentially enhance TGEV replication or the host response against the virus, during the late stages of infection.

In conclusion, we used the iTRAQ method to identify 316 significantly altered proteins in TGEV-infected ST cells. A larger number of protein expression changes occurred at 64 hpi compared to 48 hpi, indicating a larger shift in the proteome in the later stages of infection. GO analysis of these differentially expressed proteins indicated that a number of diverse biological processes are affected. In addition, many of the significant immune response related changes in protein expression we discovered are novel and, to our knowledge, have not been detected in previous proteome study. Results from this study complement the previous proteomics data obtained concerning the host response to a viral infection, and further facilitates a better understanding of the pathogenic mechanisms of TGEV infection and molecular responses of host cells to this virus.

## Supporting Information

Table S1 **Total proteins (4,364) identified and quantified by iTRAQ.**
(XLSX)

Table S2 **List of the 4,112 reliably quantified proteins selected from Table S1.**
(XLSX)

Table S3 **Differentially expressed proteins identified under different conditions.**
(XLSX)

Table S4 **GO enrichment analysis of differentially expressed proteins identified at 48 hpi.**
(XLSX)

Table S5 **GO enrichment analysis of differentially expressed proteins identified at 64 hpi.**
(XLSX)

Table S6 **GO enrichment of all the differentially expressed proteins.**
(XLSX)
